# Using the Starr Frame and Da Vinci surgery system for pelvic fracture and sacral nerve injury

**DOI:** 10.1186/s13018-018-1040-6

**Published:** 2019-01-25

**Authors:** Ye Peng, Wei Zhang, Gongzi Zhang, Xiang Wang, Shuwei Zhang, Xin Ma, Peifu Tang, Lihai Zhang

**Affiliations:** 1 Department of Orthopaedic Surgery, General Hospital of Chinese People’s Liberation Army, 28 Fu-Xing Road, Beijing, 100853 People’s Republic of China; 2 Department of Urology Surgery, General Hospital of Chinese People’s Liberation Army, 28 Fu-Xing Road, Beijing, 100853 People’s Republic of China

**Keywords:** Percutaneous pelvic fixation, Pelvic fractures, Robot-assisted, Da Vinci system, Closed reduction

## Abstract

**Background:**

Sacral fracture and sacral nerve injury remain problems in orthopedics, especially in a sacral fracture combined with an anterior sacral nerve injury. Treating a sacral nerve injury with open reduction neurolysis or more conservative treatment cannot meet the clinical needs. Open reduction sacral nerve neurolysis will increase the number of severe, life-threatening injuries, regardless of whether the anterior or posterior approach is used. In recent years, computer- and robot-assisted orthopedic surgery has emerged as part of many clinical treatments.

**Methods:**

For an unstable pelvic fracture with an anterior sacral nerve injury, we established a comprehensive and integrated solution. To achieve closed reduction, minimally invasive fixation, and minimally invasive anterior sacral nerve neurolysis, the Starr Frame, navigation robot, and Da Vinci robot were jointly applied.

**Results:**

The Starr Frame is very helpful for closed reduction percutaneous fixation in complex pelvic fractures. In this study, a minimally invasive fixation technique for the navigation robot in the pelvic fracture was explored. Although the patient had delayed anterior sacral nerve compression pain after surgery, we developed an approach and surgical method using the Da Vinci robot to explore the sacral nerve by celiac decompression. The patient was relieved of nerve pressure and pain.

**Conclusions:**

This treatment method could be an alternative treatment for pelvic fractures and sacral nerve injury. The application of this treatment is a safe and feasible option that can be employed to manage early and late nerve repair with sacral fractures when open surgery or conservative treatment is unsuitable.

## Background

The incidence of sacral nerve injury in sacral fracture ranges from 56.7 to 63.6% [1–3]. The optimal treatment of sacral fracture and sacral nerve injury remains controversial, with dissatisfactory results [4–6]. Open reduction sacral nerve neurolysis will increase the number of severe, life-threatening injuries, regardless of whether an anterior or posterior approach is used, because of the pre-sacral venous plexus and comminuted irreducible fractures in the deep pelvis.

In recent years, closed reduction and the percutaneous fixation of pelvic fractures have become increasingly popular since the introduction of the Starr Frame [7, 8]. Computer- and robot-assisted surgery has also emerged as a new and independent area in the field of orthopedics, and it has the advantages of less invasiveness, greater accuracy, and simplification of complex surgery.

However, many pelvic fractures are very complicated, especially in cases with sacral nerve injuries. It is thus challenging to achieve a good result on the basis of minimally invasive surgery using the intelligent robot technology. The combination of computers and robots with the Starr Frame for pelvic fractures has rarely been reported.

In this paper, we provide a new method for fracture reduction and percutaneous fixation as well as use of the Da Vinci surgery system for late-onset sacral nerve neurolysis. We also established a method of approach and neurolysis for this complex disease. We hope that this treatment could be an alternative strategy for use in trauma patients.

## Method

### Patient diagnosis

**Fig. 1:**
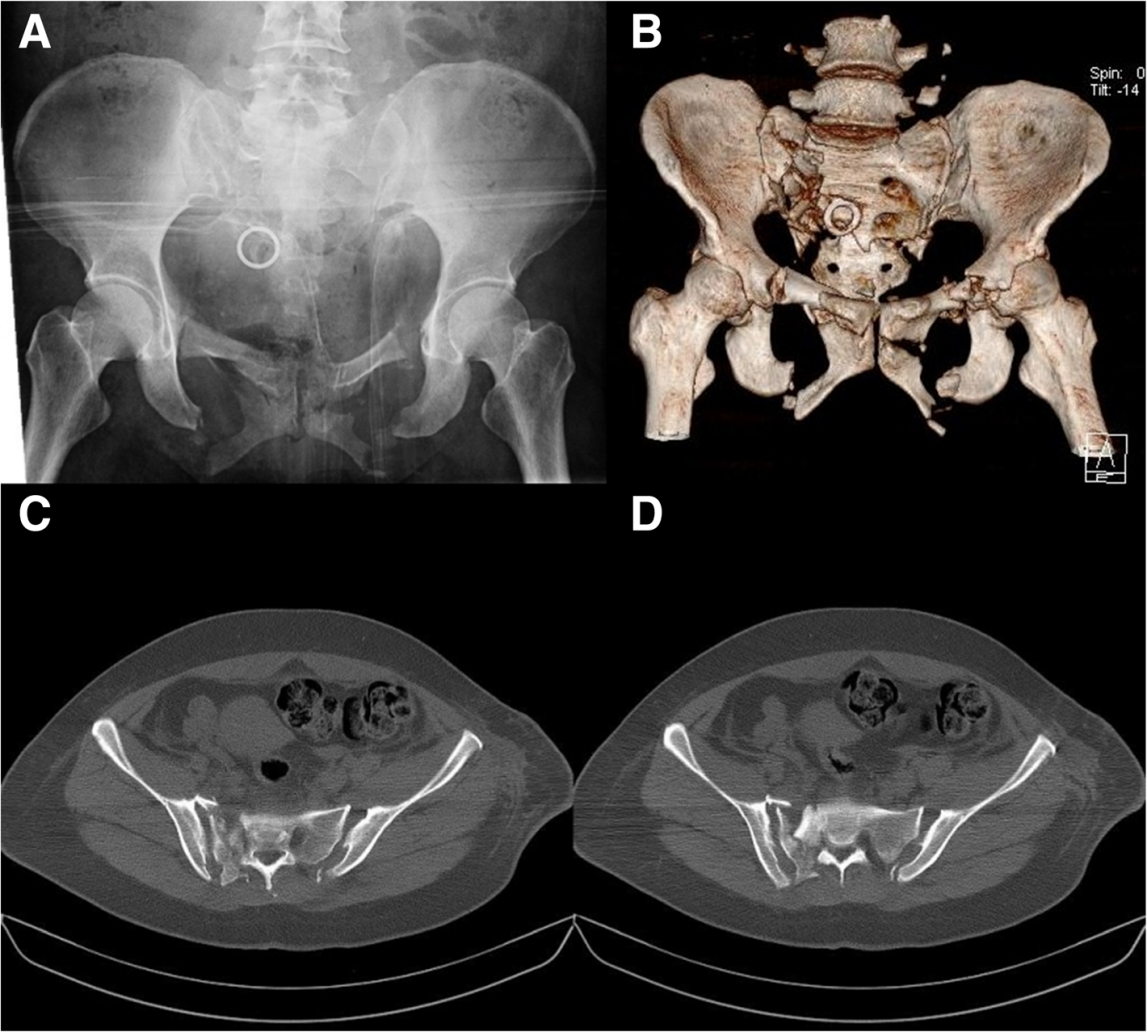
**a**–**d** AP view and CT 3D reconstruction of pelvic fracture showed type C3 (Tile classification) fracture with Denis II fracture and compressed fragment in S1 foramina

The patient was a woman with multiple injuries caused by a traffic accident. On admission, this patient was diagnosed with a pelvic fracture, bilateral pulmonary contusions, dislocation of the right shoulder, and dislocation of the left elbow. According to the Tile classification, she had a type C3 pelvic fracture, including a Denis II sacral fracture on the right side, sacroiliac joint dislocation on the left side, and bilateral pubic rami and ischial ramus fractures. Fracture fragment compression was present in the S1 sacral foramen (Fig. 1). The patient had developed numbness in the posterolateral region of the right thigh and lateral aspect of the right foot. The motor function was normal.

### Management and treatment

Her medical condition was stabilized by blood transfusion and the use of a pelvic binder. She underwent an operation 3 days after admission with the Starr Frame. We modified the Starr Frame by adding an arc stick to reduce the “open-book” injury, in which the arc center coincides with the sacrum center. We performed reduction along the arc to avoid an over-reduction or insufficient reduction of the sacroiliac joint. The pelvic ring underwent closed reduction and was fixed with the placement of a percutaneous transsacral–transiliac screw under general anesthesia.

### Follow-up and changes in illness

**Fig. 2:**
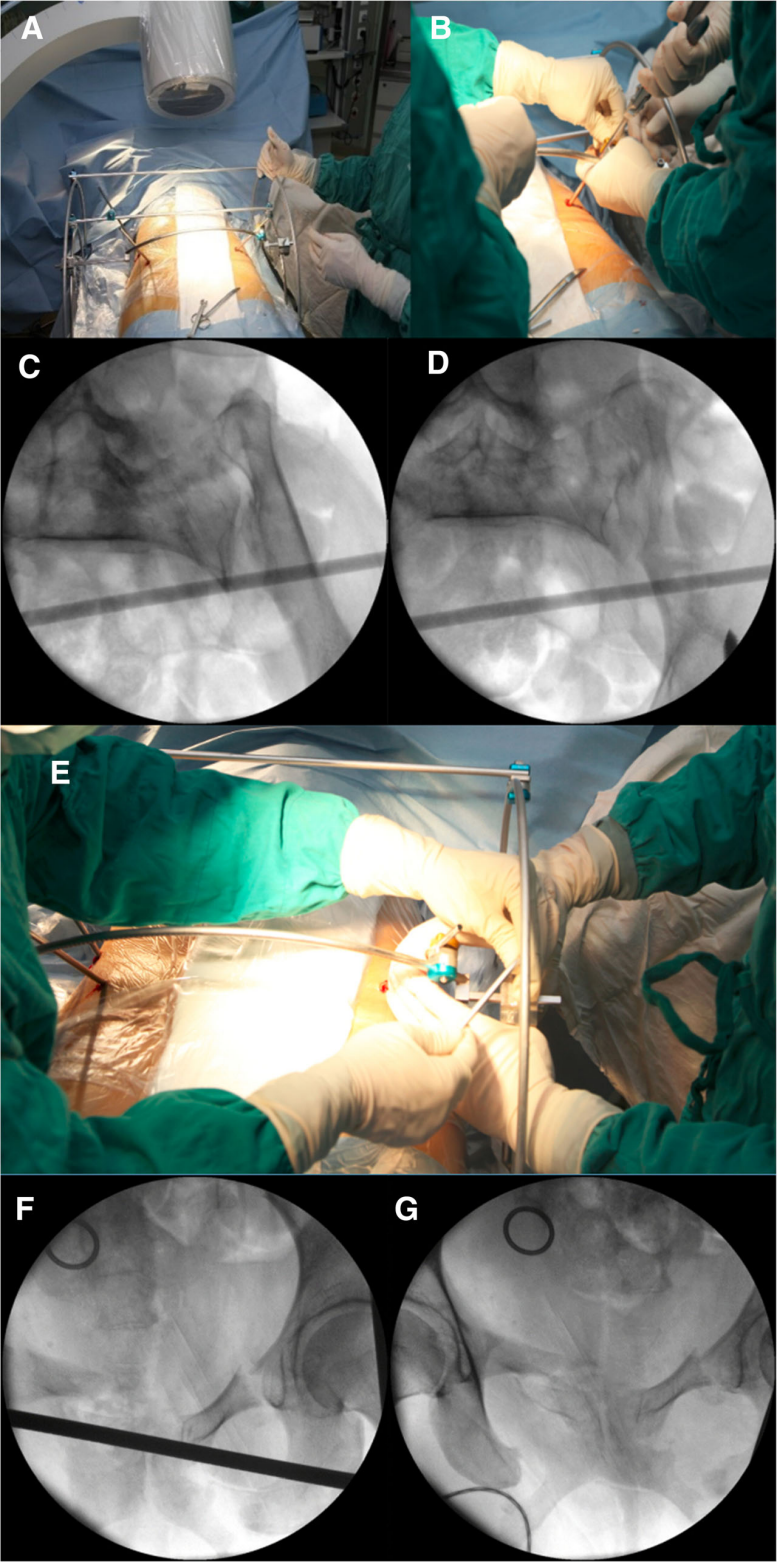
Closed reduction of pelvic fractures. **a** Starr Frame with patient. **b** Forward traction by LC-II Schanz pin. **c** Inlet view of the left sacroiliac joint before reduction. **d** Inlet view of the left sacroiliac joint after reduction. **e** Closing the LC-II Schanz pin. **f** Outlet view of the anterior pelvic ring before reduction. **g** Outlet view of the anterior pelvic ring after reduction

After the operation, the patient was in good condition and said that the numbness had been relieved in the posterolateral right thigh and lateral right foot. The post-operative inlet and outlet views of the pelvis are shown in Fig. 2. Neurotrophic agents and detumescent and analgesic drugs were used to address the patient’s symptoms.

After 7 days, the patient was discharged and was free to sit and turn over. After 4 weeks, the patient was allowed to perform weight bearing as tolerated and to start rehabilitation exercises.

At the 3-month follow-up, the patient reported neurological numbness and pain in the plantar and lateral regions of the right foot. Mecobalamin and neurotrophin were used for consecutive treatment, which effectively controlled the symptoms.

At the 2-year follow-up, the numbness and pain in the plantar and lateral regions of the right foot had worsened and were affecting the patient’s sleep. The motor function was not affected.

## Results

### Reduction strategy

**Fig. 3:**
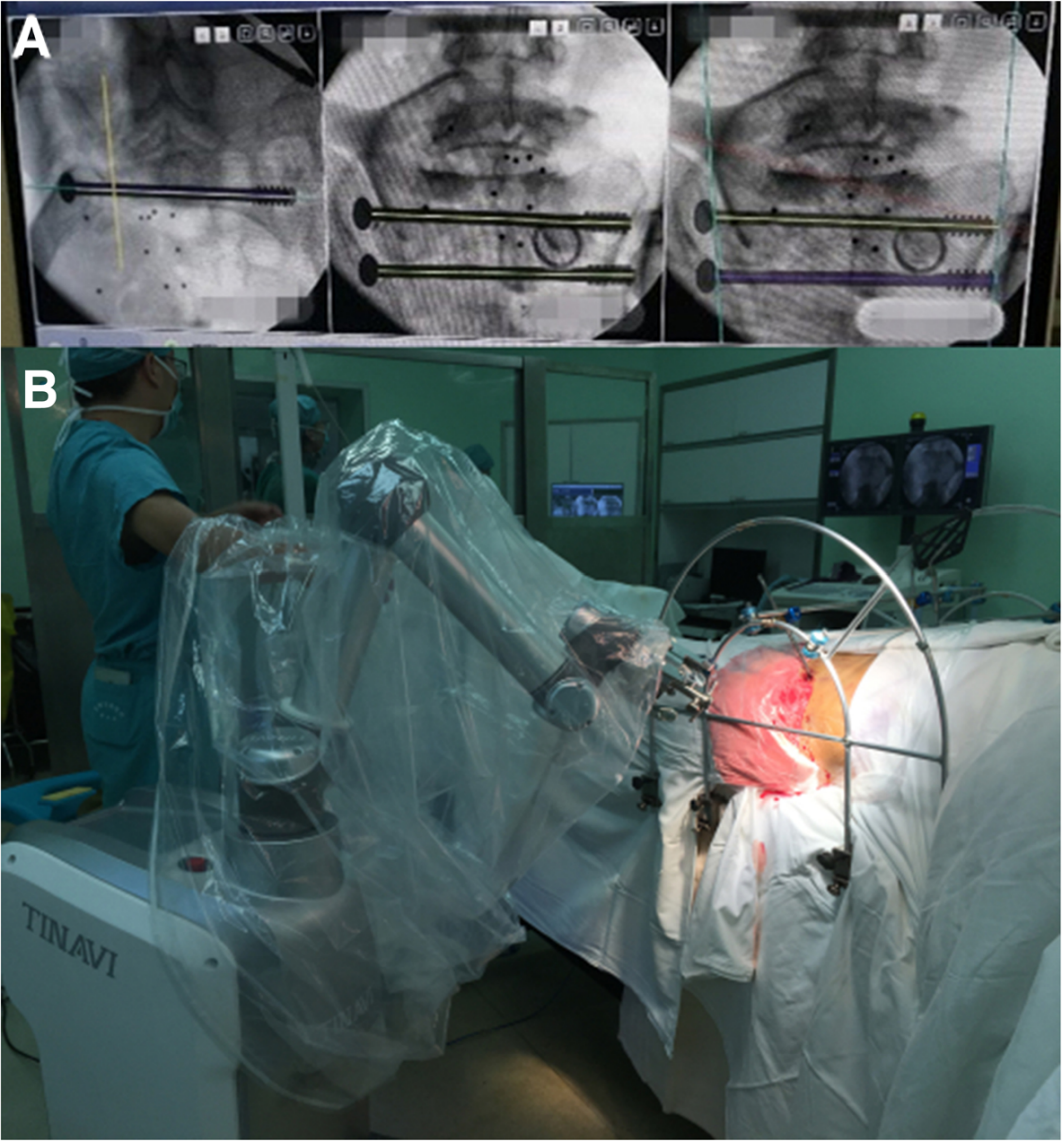
Starr Frame and computer–robot-assisted percutaneous fixation. **a** Surgical planning image of the S1 and S2 screws. **b** Starr Frame and computer–robot-assisted system

For the pelvic fracture, we reduced the posterior ring and then the anterior ring. For the posterior ring, reduction was performed with the Starr Frame in the coronal plane, sagittal plane, and transverse plane (We modified Starr Frame by adding an arc bar in the front, which the center was in the middle of the sacrum. In this way, the rotation reduction can be manipulated by moving the LC-II Schanz pin along with the arc bar and will not cause anterior or posterior displacement). There was no superior or inferior displacement in this case, so we only needed a reduction of the sagittal and transverse planes. We made forward traction by implanting a 4-mm LC-II Schanz pin in the left side to reduce the sagittal plane displacement and rotation under the inlet view. Then, we made lateral traction to reduce the out-shift of the right side sacral fragment in front of the S1 sacral foramen. Finally, the anterior ring was reduced with the LC-II Schanz pin. The reduction strategy is shown in Fig. 3.

The Starr Frame is very helpful for closed reduction percutaneous fixation in complex pelvic fracture, but it rarely works in sacral fracture segment reduction.

### Fixation strategy

**Fig. 4:**
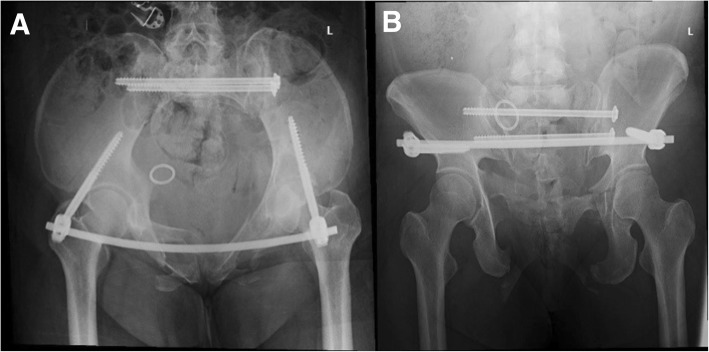
**a**, **b** The post-operation inlet and outlet views of pelvic fracture showed good reduction and fixations

The Tile C type fracture with sacroiliac joint dislocation on one side and sacral fracture on the other side required rigid fixations. The percutaneous transsacral–transiliac screw (hollow screw 7.3 mm, S1 screw length 155 mm, S2 screw length 140 mm) was implanted without compression using a computer- and robot-assisted system (TINAVI Company, China) (Fig. 4). The computer- and robot-assisted system is a 2D navigation and optical tracking system. It has control part, optical tracking part, and robot arm part. Using the assisted system, only the inlet and outlet views of the pelvis were needed. The inlet and outlet views with marker were taken and transferred to the computer systems for the surgical planning. The optical tracking system can guide the robot arm to the right position through the marker on the pelvis and inlet and outlet views with marker. The surgeon made a percutaneous screw fixation plan on the computer, and the robot arm will move the guider to the correct position for percutaneous transsacral–transiliac screw without fluoroscopy. The surgeon just needed to drill into the guide wire and implant the screw. Second, the anterior pelvic ring underwent closed reduction and internal fixation with INFIX (anterior subcutaneous pelvic fixator). The surgery took 2 h to complete and involved 200 ml of blood loss.

### Physical examination and image of CT scan

**Fig. 5:**
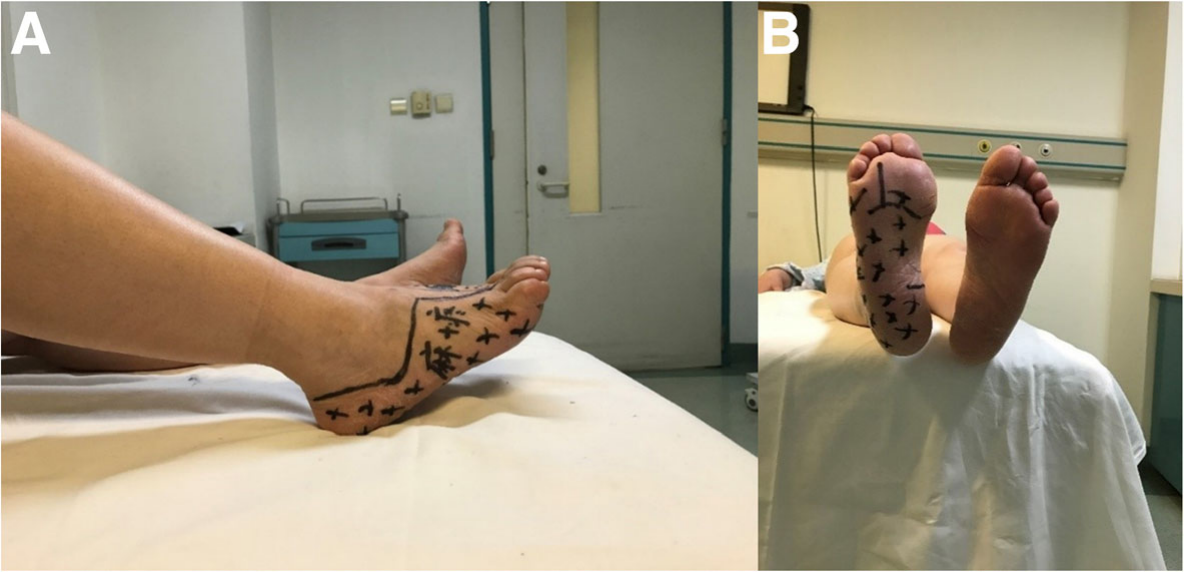
**a**, **b** The numbness and pain region on the right foot (shadow area)

The patient complained of numbness and aggressive pain of the right foot. The region is shown in Fig. 5. We examined the motor function and sensory function. The motor function is normal, but the sensory function is impaired. The electromyography examination showed nerve conduction velocity abnormal.

**Fig. 6:**
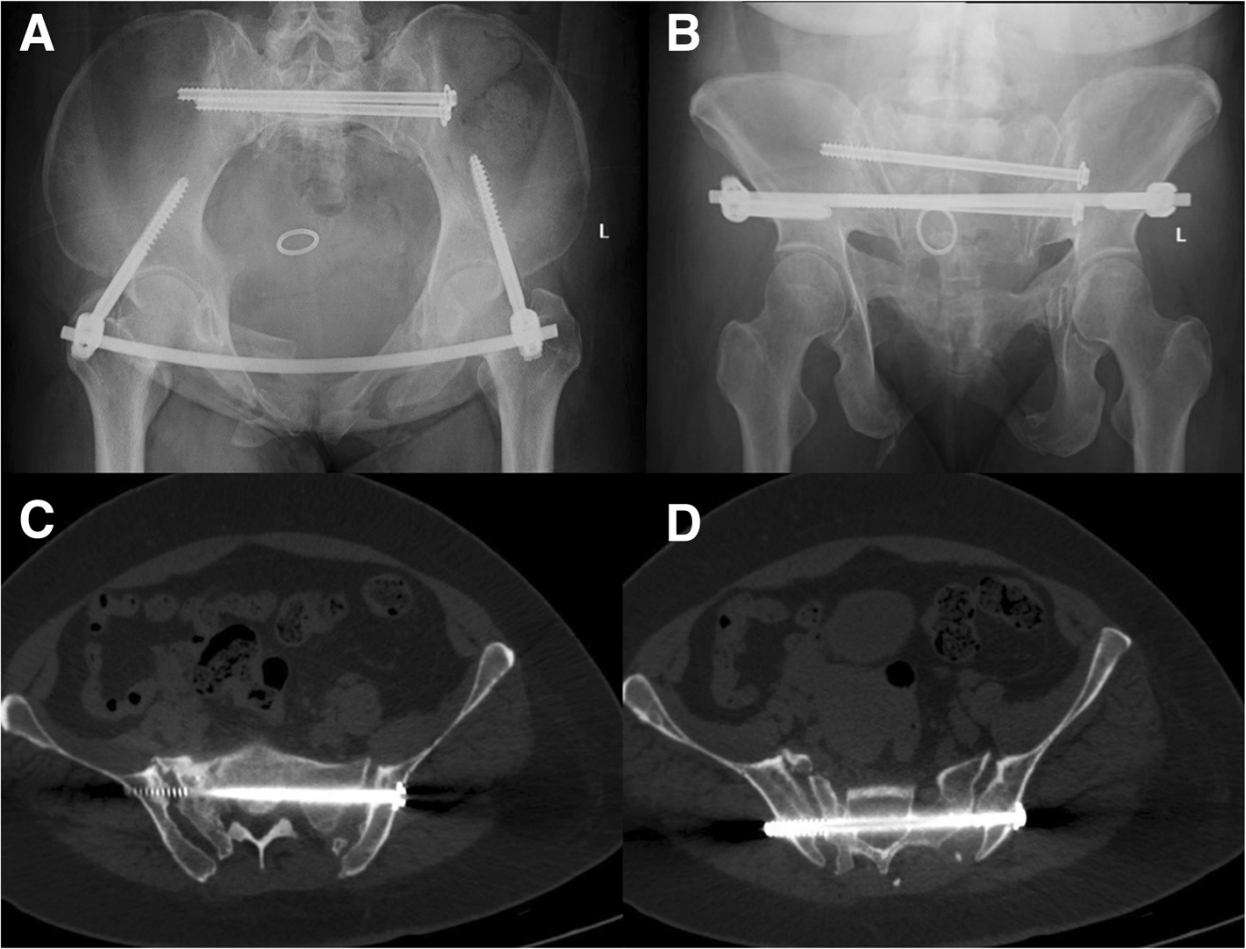
The inlet and outlet views and CT scan of the pelvis at 2 years of follow-up showed good position of screws and fracture healed. **a**, **b** Inlet and outlet views of the pelvis. **c**, **d** S1 screw and S2 screw positions of CT scan

**Fig. 7:**
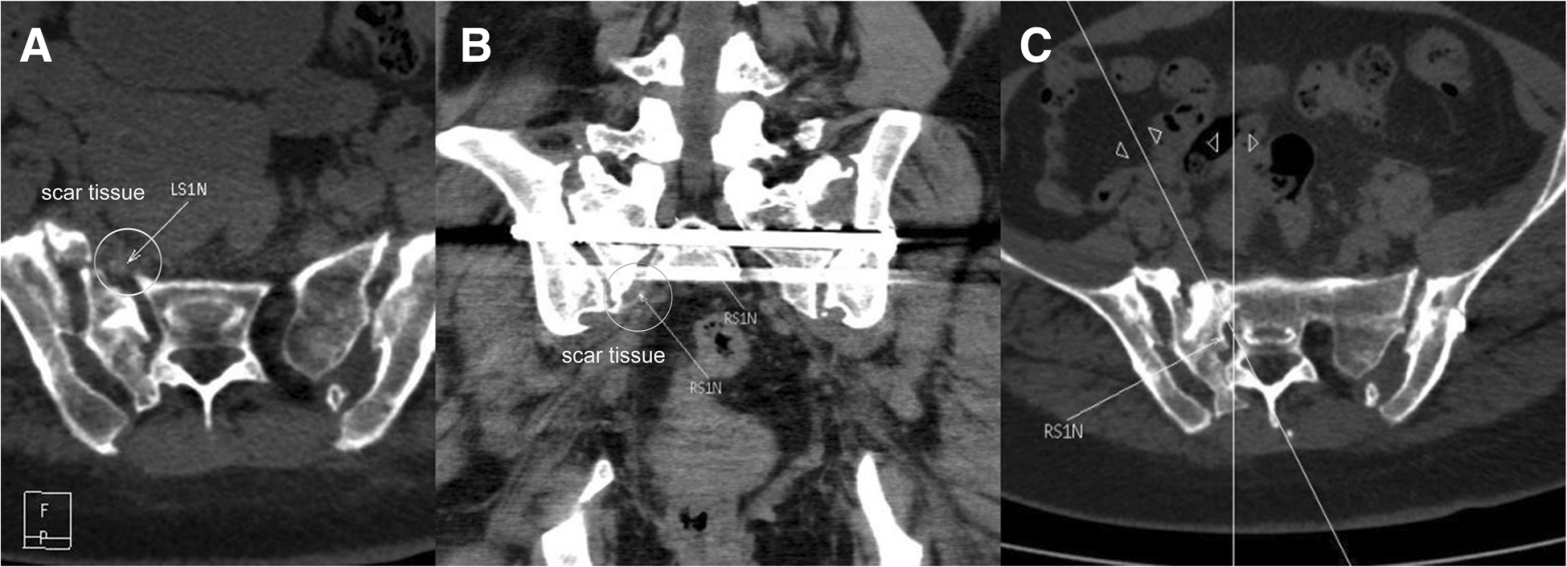
**a**–**c** The multiplanar CT reconstruction of sacral nerve showed the scar tissue (white circle) and S1 nerve (white arrow)

A pelvic CT scan showed good positioning of the transsacral–transiliac screw and a proper tunnel for the S1 nerve on the injury side (Fig. 6); however, a large amount of scar tissue was present in front of the S1 sacral foramen with multiplanar CT reconstruction nerve scan (Fig. 7).

### Neurolysis strategy

Therefore, we concluded that the scar tissue around the nerve had compressed the S1 nerve and aggravated the patient’s symptoms. Conservative treatment has been used for the incomplete sacral nerve injury about 6 months, but the symptoms were getting worse and affect the sleep of patient. The Da Vinci surgery system has been widely used for endoscopy surgery in the general surgery department and urinary surgery department. It has a control system, robot arm system, and image system. It is a minimally invasive surgery and has powerful tools for exposure, hemostasis, and release. We considered that the Da Vinci surgery system has great advantages in exploration and neurolysis of the sacral nerve and had never been reported before. So we did a neurolysis for this patient at the 2-year follow-up.

**Fig. 8:**
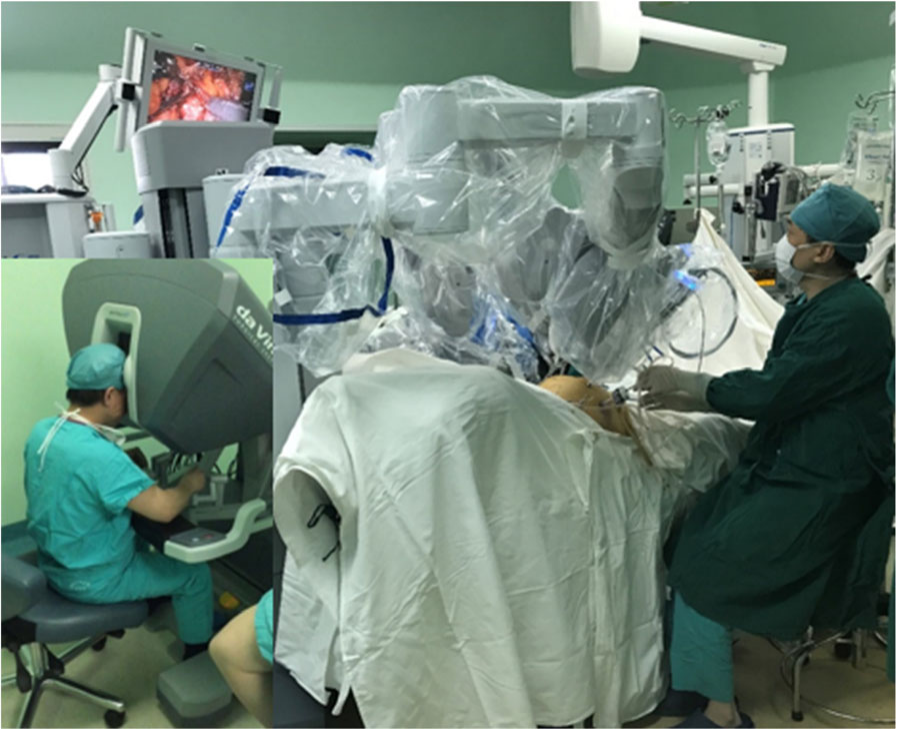
The Da Vinci surgical system for sacral nerve neurolysis

**Fig. 9:**
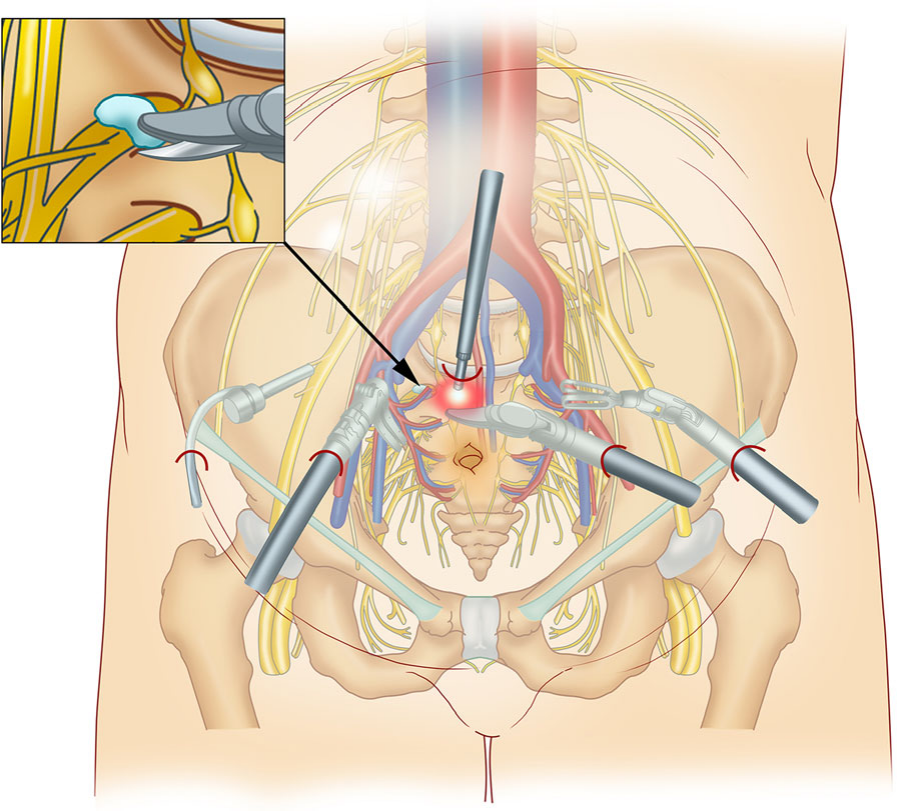
The diagram of Da Vinci surgical approach

**Fig. 10:**
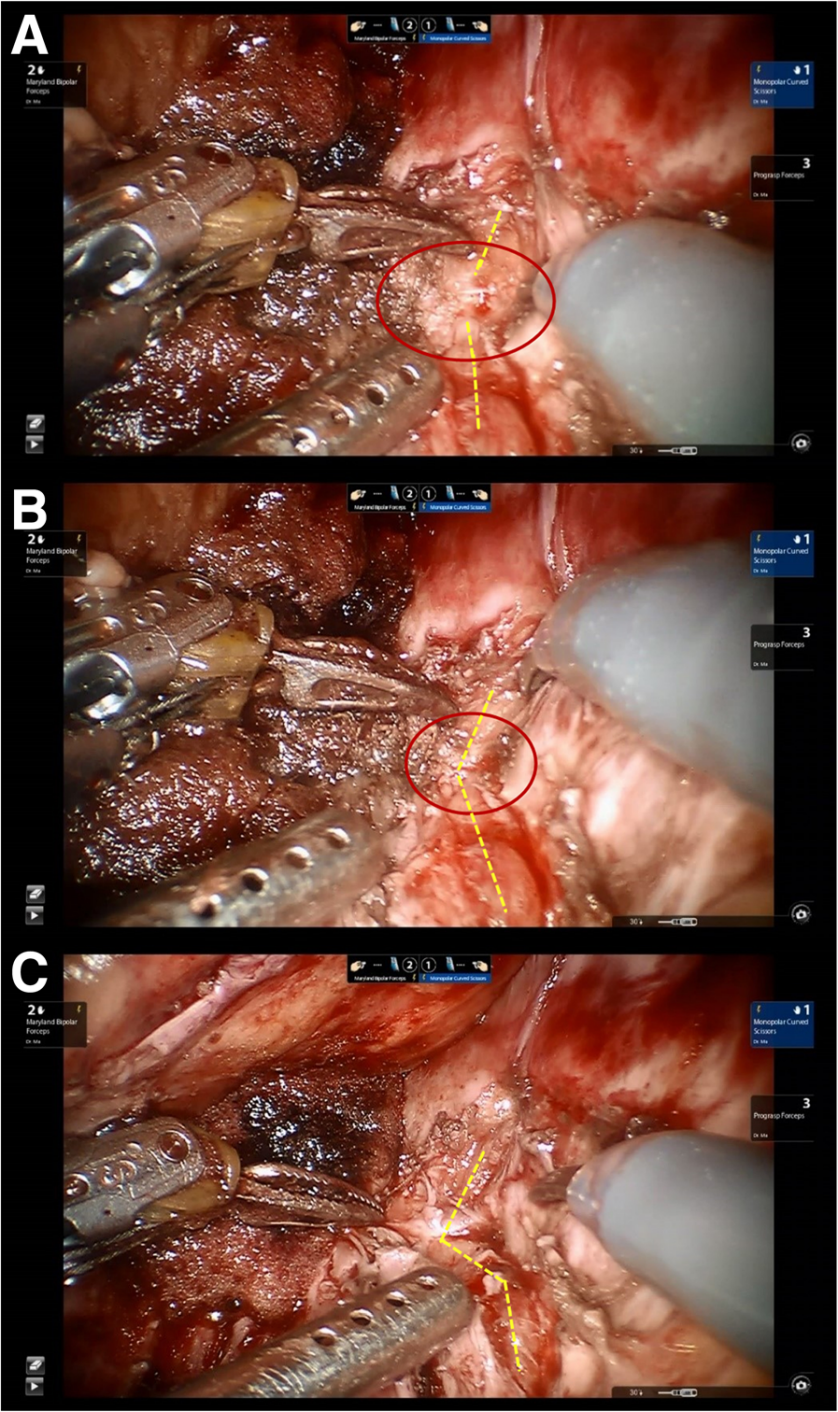
The sacral nerve neurolysis seen by the Da Vinci surgery system. **a** The scar tissue (red circle) and S1 nerve (yellow dashed line) were found. **b** Removing the adhesions and relieving the sacral nerve with monopolar curved scissors. **c** The S1 nerve without scar tissue after neurolysis

Sacral nerve exploration and neurolysis were performed with the Da Vinci surgical system under general anesthesia in the supine position (Fig. 8). The surgery approach is shown in Fig. 9. We explored the S1 nerve and found a large amount of scar tissue in front of the S1 sacral foramen. We thoroughly removed the adhesions and relieved the sacral nerve (Fig. 10). The surgery took 3 h to complete and involved a blood loss volume of 50 ml.

On the day after surgery, the patient reported that the numbness and pain had been relieved. After 3 days, the patient was discharged and returned to work. At the 1- and 3-month follow-ups, the numbness and pain had not recurred, and the patient reported good function and sensation.

## Discussion

Complex pelvic fractures with polytrauma are associated with a very high mortality rate, approximately 18%, and should thus be treated with damage control [9]. The efficacy of the Starr Frame for pelvic closed reduction and percutaneous fixation has been clinically verified [10]. However, fracture reduction using this device still has limitations. Posterior ring reduction can be easily performed by traction, rotation, and in- and out-shift manipulations, but anterior ring closed reduction is very difficult, especially for bilateral pubic rami fractures and ischial fractures. Therefore, we modified the Starr Frame by adding an arc stick in front of the pelvis. Reduction could be performed along the arc stick, avoiding an over-reduction or insufficient reduction of the sacroiliac joint or sacral fracture site upon “closing the book.” We used the Starr Frame to perform pelvic fracture reduction and transsacral–transiliac percutaneous fixation of sacroiliac joint dislocation and sacral fractures.

Many robot- and computer-assisted surgical techniques have been described [11, 12]. We used the TINAVI robot-assisted surgery system to implant two transsacral–transiliac screws, thereby decreasing the surgery and fluoroscopy times. The computer provided inlet and outlet views to calculate the best entry point for the planned percutaneous screw fixation route and then moved the guider to the correct position. The surgeon only needed to drill the guide wire along the guider and implant a percutaneous screw with an appropriate length.

The treatment of sacral fracture and sacral nerve injury remains controversial. Denis et al. [1] concluded that 6 to 8 weeks of conservative treatment has no obvious effect. Bodkin [13] reported that early decompression is necessary after confirming a fracture crush injury. Based on our experience, we believe that sacral nerve injuries should be diagnosed by multiplanar CT reconstruction and clinical examination. Early stabilization of the fracture and closed reduction of the fragments to the greatest extent possible should be performed. Conservative treatment for 3 months is suggested if the nerve is not completely ruptured. If the symptoms worsen or are unacceptable to the patient, surgical neurolysis can be performed. Unfortunately, in this case after 3 months of conservative treatment, the fractures of the sacrum were healing, which made the neurolysis more difficult to perform and caused great surgical damage.

The Da Vinci surgery system is widely used in cardiac, colorectal, general, thoracic, gynecologic, and urologic surgery [14]. It can also be used for orthopedic surgery. The Da Vinci surgery system is quite suitable for early or late decompression of a sacral nerve injury and even for the reduction of sacral fracture segments. Its advantages include easy electrocoagulation for hemostasis in the pre-sacral space, a clear view, minimal blood loss, a short surgery time, and decreased operative trauma. In this very early experience, we used the Da Vinci surgery system for sacral neurolysis.

There were some limitations to this study. First, the number of cases was limited in Da Vinci surgery neurolysis. This is our first case. As we have known, the Da Vinci surgery system for sacral nerve neurolysis had never been reported before. This study is the early experience and technique introduction. Second, Da Vinci surgery is technically demanding and has long learning curve. We believed that more and more hospital will have Da Vinci surgery system in the near future and formal training will shorten the learning curve. Finally, more comparative studies between laparoscopic surgery and Da Vinci surgery were needed for better evidence of sacral nerve neurolysis outcome.

## Conclusion

Computer- and robot-assisted surgery with Starr Frame is very helpful for closed reduction percutaneous fixation. The Da Vinci surgery system has great advantages for minimally invasive neurolysis of the sacral nerve and electrocoagulation for hemostasis in the pre-sacral space. A combination of those methods could be an alternative for pelvic fracture and sacral nerve injury. We believe that the Da Vinci surgery system and computer- and robot-assisted surgery will become more beneficial for orthopedic surgeons in the near future.

## Abbreviations

CT: Computed tomography
INFIX: Anterior subcutaneous pelvic fixator
LC-II: Lateral compression type II fracture
